# Influence of Stress on Liver Circadian Physiology. A Study in Rainbow Trout, *Oncorhynchus mykiss*, as Fish Model

**DOI:** 10.3389/fphys.2019.00611

**Published:** 2019-05-21

**Authors:** Juan Hernández-Pérez, Fatemeh Naderi, Mauro Chivite, José L. Soengas, Jesús M. Míguez, Marcos A. López-Patiño

**Affiliations:** Laboratorio de Fisioloxía Animal, Departamento de Bioloxía Funcional e Ciencias da Saúde, Facultade de Bioloxía, Universidade de Vigo, Vigo, Spain

**Keywords:** stress, liver, metabolism, clock genes, mammals, fish, rainbow trout

## Abstract

In vertebrates stress negatively affects body homeostasis and triggers a battery of metabolic responses, with liver playing a key role. This organ responds with altered metabolism, leading the animal to cope with the stress situation, which involves carbohydrate and lipid mobilization. However, metabolism among other physiological functions is under circadian control within the liver. Then, metabolic homeostasis at system level involves circadian timing systems within tissues and cells, and collaborate with each other. During chronic stress, cortisol maintains the liver metabolic response by modulating carbohydrate- and lipid-related metabolism. Stress also disrupts the circadian oscillator within the liver in mammals, whereas little information is available in other vertebrates, such as fish. To raise the complexity of this process, other candidates may mediate in such effect of stress. In fact, sirtuin1, a link between cellular sensing of energy status and circadian clocks, participates in the response to stress in mammals, but no information is available in fish. Considering the role played by liver in providing energy for the animal to deal with an adverse situation, and the existence of a circadian oscillator within this tissue, jeopardized liver circadian physiology during stress exposure might be expected. Whether the physiological response to stress is a well conserved process through the phylogeny and the mechanisms involved in such response is a question that remains to be elucidated. Then, we provide information at this respect in mammals and show comparable results in rainbow trout as fish animal model. Similar to that in mammals, stress triggers a series of responses in fish that leads the animal to cope with the adverse situation. Stress influences liver physiology in fish, affecting carbohydrate and lipid metabolism-related parameters, and the circadian oscillator as well. In a similar way than that of mammals different mediators participate in the response of liver circadian physiology to stress in fish. Among them, we confirm for the teleost rainbow trout a role of nuclear receptors (*rev-erbβ*), cortisol, and sirt1. However, further research is needed to evaluate the independent effect of each one, or the existence of any interaction among them.

## Introduction

When an animal is subjected to stress coordinated behavioral and physiological responses initiate in order to compensate and/or adapt to a new situation. However, the animal is enabled to overcome such threat. When the animal experiences intense stress, the response becomes dysfunctional, and can lose its adaptive value, thus probably resulting in inhibited growth, reproductive failure, and decreased resistance to pathogens, among other negative effects (Barton et al., 1987; Barton and Iwama, 1991). The physiological responses to a stressor are either specific for a single, or a group of related stressors (more typical), or non-specific, commonly due to different stressors. All the levels of animal organization are involved in a process known as integrated response to stress (Wendelaar-Bonga, 1997). By other hand, depending on the duration, stress can be considered as acute or chronic. Acute stressors are events that animal experiences for a short time period, for example, handling procedures. Chronic stress refers to a given situation that persists for longer time periods, thus with the physiological response of the animal being permanently activated (Tort and Teles, 2011). Among other negative effects, prolonged exposure to stress causes alterations in dendritic architecture, synapse density, and neurogenesis within the brain (Shonkoff et al., 2009). Also, gene expression can change, among other effects (for a review, see McEwen et al., 2015). Hypothalamic–pituitary–adrenal gland (HPA) activation persists during repeated stress, which leads to increased risk of depression and anxiety disorders (McEwen, 2008), as consequence of altered corticosterone system, the orexin system and others (Sargin, 2018). Whatever the case, stress may have severe negative effects on animal’s welfare.

With respect to fish, this vertebrate group have also developed mechanisms to cope with stress-induced alterations of internal homeostasis, which is indicative of the physiological stress response to be a marked adaptive character (Wendelaar-Bonga, 1997) all over vertebrates. Although the most frequent stressors in fish are those related to changes in water quality (temperature, salinity, oxygenation, pH, contaminants) and interaction with individuals of the same or different species (competition, predation, etc.) (Wendelaar-Bonga, 1997; Gesto et al., 2013), intensive fish aquaculture brought new potential stressful situations, such as high stock density, confinement, low water renovation, the presence of sick individuals, transport, altered photoperiod and feeding schedule, etc. Then welfare might be adversely affected, which influences fish growth and plant productivity.

In vertebrates, it is well described that a stressful stimulus triggers a response of two neurohormonal pathways: (1) the hypothalamic–sympathetic–chromaffin (HSC), which once activated enhances plasma catecholamines level very quickly (even seconds) and their half-life is brief (minutes); (2) the HPA, which is known as hypothalamic–pituitary–interrenal cells (HPI) in fish, and once activated, cortisol synthesis and release tend to rise slowly and remaining high when stress persists over time (chronic stress). Regarding this HPA/HPI axis, stress is responsible of glucocorticoids production and release from the adrenal gland, which is fish locates at the head kidney. Then, the hypothalamic paraventricular nucleus receives stress signals from hippocampus, amygdala and prefrontal cortex, and stimulates CRH secretion, thus activating the HPA axis. Glucocorticoids need to be newly synthesized, then delaying the response. As a consequence, slower dynamic than that of HSC is observed.

In the same way than that of other vertebrates, these axes in fish are regulated by specific biochemical factors, including adrenocorticotropic hormone (ACTH), corticotropin-releasing factor (CRF), arginine vasotocin (AVT), and brain neurotransmitters, DA, NA, and 5-HT (Winberg and Nilsson, 1993; Wendelaar-Bonga, 1997; Balment et al., 2006). This vertebrate group is also characterized by hormonal dynamics being differentially affected the type of stress, intensity, species and their previous story (Barton et al., 1987; Barton, 2002; Aluru and Vijayan, 2009; Gesto et al., 2013), which makes difficult to fully understand how stress is acting.

Among many other effects all over the body, stress hormones induce metabolic reprogramming in a tissue-specific way, in order to deal with the increase of energy demand during stress exposure, thus affecting specific tissues such as the liver. In this way, cortisol effects on metabolism have been extensively studied in mammals, with the hormone increasing glucose availability in liver through activating the major gluconeogenic pathways and the synthesis of glycogen, together with the inhibitory effect of the ability of other tissues to capture glucose (Goldstein et al., 1992, 1993; Fujiwara et al., 1996). In fish it has been described a great variety of metabolic effects of cortisol depending on the species and situations in which high levels of cortisol are generated. However, there is a consensus on the strong increase in gluconeogenesis and lipolytic potential in liver as a consequence of the stress-induced increase of plasma cortisol levels (Vijayan et al., 1991, 1994; López-Patiño et al., 2014b). It is also well known that cortisol metabolic action in fish is mediated by glucocorticoid receptors (GRs), which in most teleost fish display two isoforms (GR1 and GR2) that locate in a large number of central and peripheral tissues (Ducouret et al., 1995; Teitsma et al., 1997, 1998; Jaillon et al., 2004).

By other hand, metabolism, together with many other physiological functions is under circadian control in all living organisms in such a way that metabolic homeostasis at system level needs the timed collaboration of cells and tissues all over the body. Such rhythms are driven by cell autonomous clocks at central and peripheral locations, which synchronize the organism to the environmental cycles, even in the absence of environmental cues (Hardin and Panda, 2013). Clocks molecular mechanism is highly conserved in phylogeny and although with some differences, it is easily identifiable in the different organisms. The basic mechanism involves a series of feedback loops between the transcription and transduction processes of certain genes (called “clock genes”) and their protein products (Panda et al., 2002). The most known model determines that the system initiates with the accumulation of cytoplasmic CLOCK and BMAL1 proteins as the products of *clock* and *bmal1* transcription. These proteins form a heterodimer (CLOCK/BMAL1) that returns to the nucleus, joining E-Box promoters in the target genes, including the negative branch genes of the loop: *per* and *cry*. Their transcription results in increased cytoplasmic levels of their protein products, PER and CRY, so that when the levels are high enough they dimerize, thus inhibiting the CLOCK/BMAL1 complex function (Kondratov, 2007).

Mammalian circadian system is the most studied so far, and the suprachiasmatic nucleus (SCN) is considered the main pacemaker. Then, the SCN hosts the master oscillator containing specific molecular elements (clock genes) that create/control circadian rhythms of most functions all over the organism. In support of this, behavioral, endocrinological, and physiological circadian variations disappear in lesioned SCN (Moore et al., 1995). SCN receives information mainly through three different pathways: retinohypothalamic tract (RHT), intergeniculate leaflet (IGL), and medial nucleus of the rafe (RM). RHT pathway originates at the retina and is the main photic-related input, thus playing a critical role in generating circadian rhythms (Weaver, 1998; Gooley et al., 2001), whereas the other pathways participate as inputs of photic information to the suprachiasmatic nucleus (IGL) or non-photic information (RM). In addition, rhythms of clock genes expression has been reported in tissues other than the SCN, such as liver, muscle, adipose tissue, pancreas, kidney, lung, and ovary (Balsalobre, 2002; Mühlbauer et al., 2004; Peirson et al., 2006; Zvonic et al., 2006), which is indicative of the existence of circadian oscillators in them all.

In non-mammalian vertebrate groups multiple coupled central circadian oscillators exist. These oscillators can locate within different tissues such as retina, pineal gland and hypothalamus, but their functioning appear to remain quite similar to that of mammals (Menaker et al., 1997; Falcón et al., 2010; López-Patiño et al., 2011). In addition to hypothalamus in fish, retina and pineal organ also host circadian oscillators, since both tissues are photosensitive, clock genes express rhythmically within them, and release an endocrine output, such as the melatonin (Falcón, 1999; Falcón et al., 2007). In the same way than that of mammals, clock genes rhythmically express in peripheral tissues of non-mammalian vertebrates, such as liver, heart, intestine, and muscle of birds (Chong et al., 2003), liver, heart, muscle, lung, and testis of reptiles (Della Ragione et al., 2005; Vallone et al., 2007), and liver, heart, spleen, and gall bladder of fish (Kaneko et al., 2006; Velarde et al., 2009; Betancor et al., 2014; Hernández-Pérez et al., 2017).

Specifically for fish, the circadian system is typically composed by multiple oscillators located throughout the body, and mainly entrain to external inputs, LD and feeding-fasting cycles, among others. Coordination among them all leads for successful control of different rhythmic functions (locomotor activity, hormonal rhythms…). The synthesis and release of specific outputs allow them to adjust all these functions.

One of the peripheral locations described for the circadian system is the liver. In fish, some reports describe rhythmic expression of clock genes within this tissue in different species such as goldfish (*per* and *cry*, Velarde et al., 2009), Atlantic salmon (*bmal1*, Betancor et al., 2014) and rainbow trout (*clock, bmal1, per* and *rev-erbβ*, Hernández-Pérez et al., 2017). Interestingly, such rhythms perfectly fit with those of metabolism-related parameters within this peripheral location (Polakof et al., 2007; Betancor et al., 2014; Paredes et al., 2014; Hernández-Pérez et al., 2015).

Independently of where the oscillatory machinery is located, its functioning can be disrupted by different factors. Among them, stress is the most studied so far. Accordingly, glucocorticoids and epinephrine synchronize circadian tissue clocks (Balsalobre et al., 2000; Akiyama et al., 2003; So et al., 2009), but stress affects the circadian system in many other ways. For example, the effectors of the stress response exert their action through specific receptors. Regarding cortisol as the main glucocorticoid, the hormone displays daily rhythms of plasma levels and binds to either GRs or mineralocorticoid receptors (MR), with the latest binding to cortisol even at the time of the day in which plasma glucocorticoid levels are in the minimum. This makes MR signaling pathway not to be effective in conveying time-of-day information (see rev. Koch et al., 2017). The other mediator, GR, is expressed all over the organism (Kino and Chrousos, 2004; Chrousos and Kino, 2005; Nader et al., 2010), but not at the SCN, which makes this tissue not to get any synchronizing feedback through GRs in vertebrates (Balsalobre et al., 2000), whereas no information is available at this respect in other groups such as fish.

Regarding GR signaling, classical and non-classical pathways are reported (Beato et al., 1987; Freedman, 1992; Groeneweg et al., 2012) and contribute to a high level of complexity. Among the classical ones, the interaction of GR dimers with glucocorticoid response elements within regulatory regions of GC target genes is the most studied so far, and such elements are identified in some clock genes (So et al., 2009). Transcription of target genes can be also activated by other transcription factors, nuclear factor-κB (NF-κB), activator protein-1 (AP-1), or STAT5 (Scheinman et al., 1995; Garside et al., 2004; Kino and Chrousos, 2004; Chrousos and Kino, 2005; De Bosscher and Haegeman, 2009). Additionally, GR binding to negative glucocorticoid response elements mediates the *trans*-repression of negatively regulated genes (Surjit et al., 2011). The interaction of GRs with DNA can influence surrounding DNA-bound transcription factors as well (Groeneweg et al., 2012; Samarasinghe et al., 2012). The activation of one of these pathways takes place in minutes to hours. On the contrary, the non-classical signaling is independent of transcription and gene expression (Groeneweg et al., 2012), thus resulting in a faster response (seconds to minutes). Altered activity of some kinases (phosphoinositide 3-kinase, PI3K; AKT, and mitogen-activated protein kinases, MAPKs) is responsible of this pathway.

The influence of stress on circadian rhythms has been addressed in rodents. Accordingly, clock genes phase advance in peripheral organs of acutely stressed mice during the early day (Tahara et al., 2015). On the contrary, stress exposure at different time of the day causes a phase delay or the loss of synchrony. This is indicative of the time of the day-dependence of the influence of stress on peripheral clocks. However, no changes were observed within the SCN, where GRs do not express in this vertebrate group, but chronically repeating a given procedure, such as social defeat results enhances the amplitude of *Per2* rhythm within the SCN (probably as consequence of activated indirect mechanisms), and downregulates *Per2* and *Cry1* expression within the adrenal gland of animals stressed at the early dark phase, whereas stress the early day phase advances the adrenal oscillator but has no effect on the SCN clock (Bartlang et al., 2014).

Studies at this respect in other vertebrate groups such as fish are scarce. In this way, goldfish receiving a cortisol administration display similar results than those observed in mammals, with inhibited expression of some clock genes within the liver (Sánchez-Bretaño et al., 2015). However, no evidence exists relative to how stress affects rhythmic physiology within this tissue in this vertebrate group, but recent results indicate altered metabolism-related parameters within rainbow trout liver following acute stress (López-Patiño et al., 2014b), where a circadian oscillator has been reported to exist in the same species (Hernández-Pérez et al., 2015, 2017).

On the other side, chronic or repeated exposure to a stressor makes the body to adapt, resulting in altered functions such as energy metabolism, which may raise the incidence of metabolic disorders, as reported for humans and rodents (see rev. Koch et al., 2017). Even when it is of high clinical interest to address the impact of social stress on circadian functions, it is likely that available data are not sufficient, since many studies do not report the time of stress exposure, and just few compare the impact of the stressor all over the day. Also, stress response is dependent of the stressor (Gattermann and Weinandy, 1996), which makes difficult to compare results among studies. Depressive and anxiety-related behaviors are reported for rat subjected to chronic mild stress applied only during the light phase (Aslani et al., 2014). Also, other stressors (cat smell, tail shock, and immobilization) are more effective when applied when animals are in their phase of inactivity (Retana-Marquez et al., 2003; Cohen et al., 2015; Fonken et al., 2016).

Regarding fish, chronic stress might negatively affect the circadian system, followed by the alteration of rhythmic behavioral and physiological functions. In fact, endocrine rhythms are outputs of the circadian system, whereas some hormones may play a role as inputs to the circadian system in hypothalamic and peripheral oscillators (Challet, 2015; Coomans et al., 2015). Among them, glucocorticoids, as stress response mediators, display daily rhythms in fish (in the same way than other vertebrate groups), and cortisol rhythm in fish synchronizes to feeding fasting cycle and feeding time (see rev. Isorna et al., 2017). This is indicative of the hormone to be an output of the circadian system, thus being under circadian control. Accordingly, daily variations of plasma cortisol was reported for different fish species (Cerdá-Reverter et al., 1998; Pavlidis et al., 1999; Small, 2005; Ebbesson et al., 2008) and even related with feeding and metabolism, suggesting a possible synchronization of the cortisol secretion with feeding time (Spieler and Noeske, 1984). However, it is not defined whether cortisol is an input to the circadian system in fish or not. It is reported that cortisol stimulates the *per1a* and *per1b* expression, and inhibits that of *clock* and *bmal1* in liver of goldfish (Sánchez-Bretaño et al., 2016). Then, cortisol could mediate the effects of stress on liver circadian physiology in fish. Our preliminary results in rainbow trout point to such role, since liver of mild-stress animals shows decreased amplitude of *clock1a* and *bmal1* mRNA abundance daily rhythms, together with altered *per1* rhythm (unpublished data). Then, glucocorticoids may play a key role as mediators of the altered functioning of the circadian system observed in stressed fish.

Taking into account the interactions between cortisol, metabolism and circadian system, it is possible that the alteration of cortisol levels as a consequence of stress can trigger effects on liver metabolism and circadian system in fish, as in mammals. Accordingly, our aim was to elucidate how stress affects gene expression of clock- and metabolism-related genes, and to corroborate if metabolic response to chronic stress exposure results from altered circadian machinery within the liver of rainbow trout (*Oncorhynchus mykiss*), in the same way than that reported for other vertebrate groups.

## Materials and Methods

### Animals

Rainbow trout (*Oncorhynchus mykiss* Walbaum) of 94 ± 8 g of body weight were transferred to our facilities (Faculty of Biology; Vigo, Spain) from a local hatchery (A Estrada, Spain). Animals adapted to our laboratory conditions for at least 15 days before any experiment was performed. Then, fish remained in 120 L tanks (10 kg of fish/m^3^) with filtered and continuous water renovation (13.5 ± 1°C). Food consisted on a commercial (Dibaq Diproteg, Segovia, Spain) dry pellet diet (1% body weight), and was provided at zeitgeber time (ZT/CT) 2. Trout were kept in a 12:12 LD photoperiod, with lights on at ZT0. Light intensity was 500 lux at the water surface during photophase, and did not exceed 0.3 lux at scotophase. Experiments comply with European Union Council Guidelines (2010/63/EU), and Spanish Government (RD 53/2013) for the use of animals in research. Also, the Animal Care Committee at the Vigo University approved all the animal protocols, following the international ethical standards (Portaluppi et al., 2010).

### Sampling

Fish were deeply anesthetized with MS-222 (50 mg L^-1^) buffered to pH 7.4 with sodium bicarbonate, and weighed. Animals were sacrificed and sampled every 4 h during a 24-h light/dark cycle, starting at ZT0 (lights on). Accordingly, samplings time points were ZT0, ZT4, ZT8, ZT12, ZT16, ZT20, and ZT0′ on the following day. Time needed for sacrifice and sampling procedures was never longer than 15 min/time point. Once deeply anesthetized, caudal puncture with ammonium heparinized syringes was performed for individual blood collection. Fish were sacrificed immediately after, and liver from each animal was dissected and divided into two portions under sterile conditions. Each portion was placed in sterile RNase-free 1.5 mL Eppendorf tubes, and immediately frozen in liquid nitrogen, and stored at -80°C until assayed for metabolites content, enzyme activities, and gene expression quantification. Blood was centrifuged to obtain plasma samples that were immediately frozen on liquid nitrogen and stored at -80°C until assayed for cortisol, glucose and lactate levels.

### Experimental Design

To evaluate the impact of stress on liver rhythmic physiology in rainbow trout, two experimental groups of fish (*N* = 56) were randomly distributed in seven tanks each, which were initially acclimated to standard lighting conditions (L:D 12:12) for at least 2 weeks. After that, one of the groups of fish remained in the same condition (Control, C), while the second one was submitted to high density stress (Stress, ST), by reducing water level of the tanks up to reach a density of 70 kg of fish/m^3^ (Conde-Sieira et al., 2014). Fish remained 72 h in these experimental conditions, being fed at the same time as during the acclimatization phase (ZT2). On the last day fish were captured from each tank (control and stress groups) and sampled as mentioned above.

### Assessment of Cortisol Levels and Liver Enzyme Activities

The Enzyme Immunoassay Kit (Cayman, Ann Arbor, MI, United States) was purchased for plasma cortisol assessments, following manufacturer’s specifications. A portion of liver was assessed for enzyme activities. Then, homogenization by ultrasonic disruption was performed for each, in 10 vol. ice-cold buffer: 50 mmol L^-1^ Trizma (pH 7.6), 5 mmol L^-1^ EDTA, 2 mmol L^-1^ 1,4-dithiothreitol, and a protease inhibitor cocktail (Sigma P-2714). The homogenate was centrifuged (10 min at 10,000 × *g*) and the supernatant collected for enzyme assays. The INFINITE 200 PRO microplate reader (Tecan, Grödig, Austria) was used for enzyme activities determination. The reaction rate of each enzyme was determined by the increase/decrease of NAD(P)H absorbance at 340 nm. Reactions initiated after addition of homogenates (15 μL), at a pre-established protein concentration, omitting the substrate in control wells. Then, reactions did proceed at 20°C for 3–10 min. Enzyme activities were expressed as relative to mg of protein. Then, protein content in each homogenate was assayed in triplicate following the bicinchoninic acid method with BSA (Sigma, Saint Louis, MO, United States) as standard. Enzymatic analyses were performed at maximum rates, with the reaction mixtures set up in preliminary tests to render optimal activities. GK, PEPCK, G6Pase, PK, HOAD, and FAS activities were evaluated as described previously (Polakof et al., 2007, 2008; Librán-Pérez et al., 2012).

### Real-Time Quantitative RT-PCR (qPCR)

The TRIzol^®^(Life Technologies, Grand Island, NY, United States) method was used for total RNA extraction in individual rainbow trout liver. The extract was mixed with RQ1-DNAse (Promega, Madison, WI, United States). Then, 2 μg of RNA from each sample was reverse transcribed into cDNA, for which M-MLV reverse transcriptase (Promega) and Random Primers (Promega) were used. To discard genomic contamination of the RNA extract a negative control of each sample was assessed in the absence of reverse transcriptase.

To perform the qPCR, Maxima^TM^ SYBR Green qPCR Master Mix (Thermo Scientific, Waltham, MA, United States) and the Bio-Rad MyIQ Real Time PCR system (BIO-RAD, Hercules, CA, United States) were used. All primers and probes (Table 1) were designed according to existing sequences for rainbow trout genes, and obtained from Sigma.

**Table 1:** Primers’ sequences, forward (F), and reverse (R) of different genes measured along the study, with the specific annealing temperature and reference for each gene.

Gene	Sequence	Annealing T^a^	References
*gk*	**F:** *GCACGGCTGAGATGCTCTTTG*	60	AF053331 (GenBank)
	**R:** *GCCTTGAACCCTTTGGTCCAG*		
*pk*	**F:** *CCATCGTCGCGGTAACAAGA*	59	AF246146 (GenBank)
	**R:** *ACATAGGAAAGGCCAGGGGC*		
*pepck*	**F:** *GTTGGTGCTAAAGGGCACAC*	59	AF246149 (GenBank)
	**R:** *CCCGTCTTCTGATAAGTCCAA*		
*g6pasa*	**F:** *CTCAGTGGCGACAGAAAGG*	55	cay0019b.d.18_3.1.s.om.8.1-1693 (Sigenae)
	**R:** *TACACAGCAGCATCCAGAGC*		
*GLUT2*	**F:** *GTGGAGAAGGAGGCGCAAGT*	59	AF321816 (GenBank)
	**R:** *GCCACCGACACCATGGTAAA*		
*fas*	**F:** *GAGACCTAGTGGAGGCTGTC*	59	tcab0001c.e.06 5.1.s.om.8 (Sigenae)
	**R:** *TCTTGTTGATGGTGAGCTGT*		
*hoad*	**F:** *GGACAAAGTGGCACCAGCAC*	59	Tcad0001A.I.15_3.1.om (Sigenae)
	**R:** *GGGACGGGGTTGAAGAAGTG*		
*GR1*	**F:** *AGAAGCCTGTTTTTGGCCTGTA*	59	NM_001124730.1 (GenBank)
	**R:** *AGATGAGCTCGACATCCCTGAT*		
*GR2*	**F:** *CATCGCAGACCAGTCTGAAC*	55	AY495372.1 (GenBank)
	**R:** *AGCAGCAGCAGAACCTTCAT*		
*clock1a*	**F:** *CTCAAGACGAAAAACCAGTTAGAA*	57	AF266745 (GenBank)
	**R:** *AGGCTCTTTGGGGTCGAT*		
*bmal1*	**F:** *TGGACATTTCCTCCACGATG*	55	GQ489026.1 (GenBank)
	**R:** *TCTTGTCCCTGCGTCTCTTC*		
*per1*	**F:** *AAGTCGTAGAGGAAGACCCA*	55	AF228695 (GenBank)
	**R:** *ATCTGTCTGCACATACCGCT*		
*rev-erbβ*	**F:** *AGCAGTGCCGCTTCAAGA*	56	AF342943.1 (GenBank)
	**R:** *CGGCCAAACCTAACAGAGTC*		
*sirt1*	**F:** *GCTACTTGGGGACTGTGACG*	55	EZ774344.1 (GenBank)
	**R:** CTCAAAGTCTCCGCCCAAC		
*b-actin*	**F:** *GATGGGCCAGAAAGACAGCTA*	55	NM_001124235.1 (GenBank)
	**R:** *TCGTCCCAGTTGGTGACGAT*		

Relative quantification of each gene transcript was assessed and *β-actin* expression was selected as housekeeping, since it does homogeneously express through the 24-h cycle independently of the experimental condition. Thermal cycling initiated with 3 min incubation at 95°C; followed by 40 steps of PCR (heating for 10 s at 95°C for denaturing, and specific annealing for 30 s and extension at 50°C for 30 s). After the last PCR cycle, melting curves were monitored (50°C temperature gradient at 0.5°C/s from 50 to 95°C) to corroborate that only one fragment was amplified. Relative mRNA expression level was calculated using the standard comparative delta-Ct method. Relative quantification of each gene transcript with the *β-actin* reference gene transcript was evaluated according to the Pfaffl method (Pfaffl, 2001). For each gene, samples from the same time point were processed in parallel, and expression was assessed in triplicate within the same microplate. Only efficiency values ranging from 85% to 100% were accepted (*R*^2^ for each gene was always higher than 0.985).

### Statistical Analysis

To determine the existence of significant differences of gene expression between time points within a given experimental condition (C and ST) and gene, the one-way ANOVA analyses were carried out, followed by the Student–Newman–Keuls test for multiple comparisons. Also, the rhythm of expression for each gene was analyzed by fitting periodic sinusoidal functions to the gene expression levels across the sampling time points using the formula *f(t)* = *M* + *Acos(tπ*/*12 -* φ*)*. Thus, *f(t)* reflected gene expression level at a given time point, the mesor (*M*) was the mean value, *A* was the sinusoidal amplitude of the oscillation, *t* was time in hours, and φ was time of the peak (acrophase). Non-linear regression allowed to estimate *M, A*, and φ, and their standard error (SE) (Delgado et al., 1993). For the sinusoidal function, all parameters were expressed as the value ± standard error (SE). The SE based on the residual sum of squares in the least-squares fit. A rhythm of expression was consistent only if either *P* < 0.05 from the ANOVA test, and *SE(A)*/*A* < 0.3 provided by the cosinor analysis, according to the principle of a noise/signal ratio less than 0.3, with “signal” being the amplitude and “noise” its error (Halberg and Reinberg, 1967).

## Results

### Plasma Metabolite Levels

**FIGURE 1:**
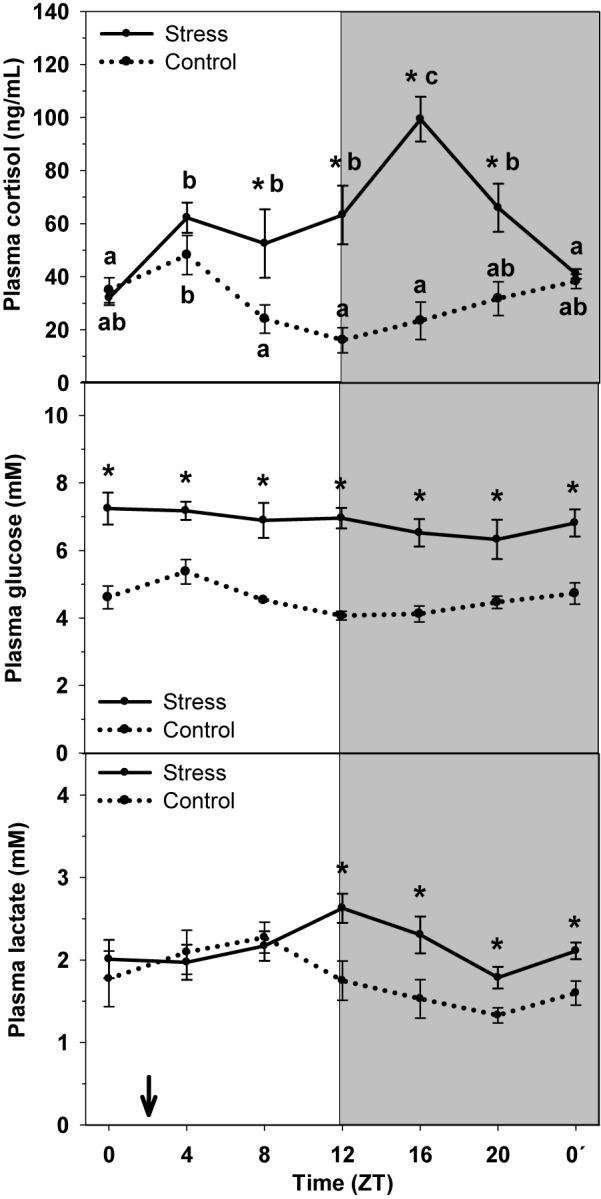
Daily profile of plasma cortisol, glucose and lactate plasma levels in control (dashed line) and stress (continuous line) rainbow trout. Each value is the mean ± SEM (*n* = 8/group). ^∗^*P* < 0.05 relative to Control at the same time point. Different letters indicate significant differences (*P* < 0.05) between time points within the same experimental group. Gray band indicates the dark phase of the daily cycle. The arrow indicates feeding time (ZT2).

Cortisol, glucose, and lactate plasma contents are represented in Figure 1. In Control, cortisol levels were highest at the early light period (ZT4) and basal levels were observed during the light-dark transition (ZT12), when started to rise slowly. Stress by high stocking density enhanced cortisol levels, which resulted in a significant raise of mean cortisol levels (58.53 ng/mL) relative to that of Control (29.01 ng/mL), and altered rhythmic profile of the hormone. Then stressed trout displayed peaking cortisol levels during the night (ZT16) and basal levels at the very end of the dark phase (ZT0′).

Plasma glucose levels showed a daily oscillation in control animals, with higher values at day-time (ZT4) and basal levels at the night onset (ZT12). Stress caused an increase in the averaged levels (6.86 mM), compared to Control (4.60 mM), but also the daily oscillation to disappear.

Lactate levels displayed a similar daily oscillation that that of glucose, with higher values being observed in samples collected at ZT8, and basal levels during the late night period (ZT20). Stress exposure resulted in the increase of averaged lactate levels (2.14 mM) compared to that of control group (1.77 mM), but also the daily oscillation changed, thus with the highest levels occurring at the day–night transition (ZT12), i.e., with a 4-h delay.

### Liver Metabolite Levels

**FIGURE 2:**
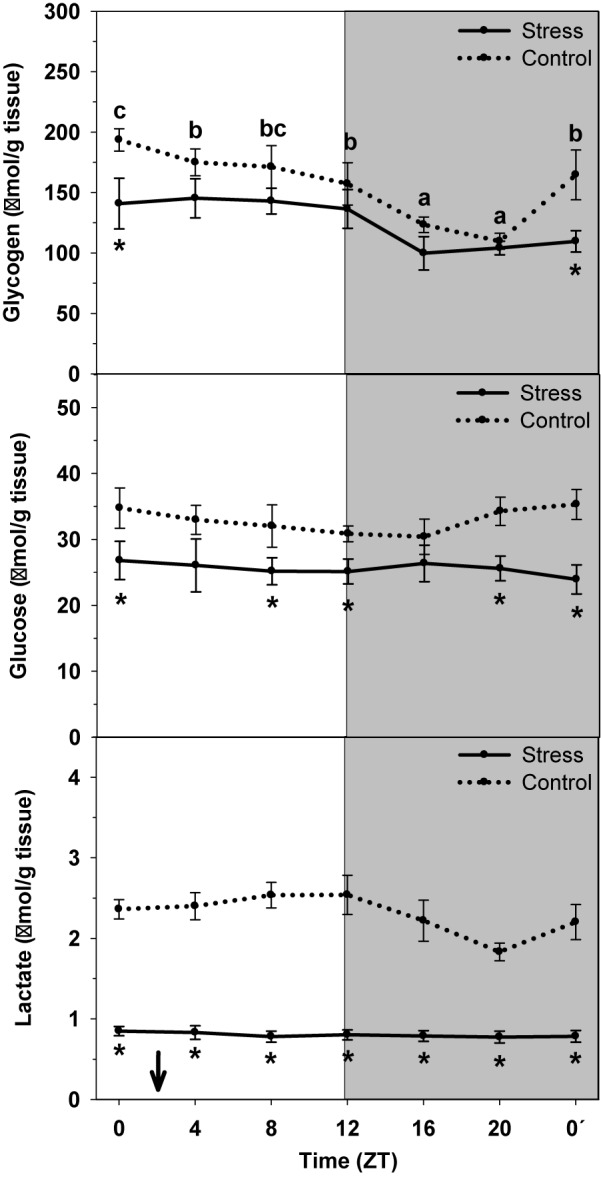
Daily profile of glycogen, glucose and lactate liver levels in control (dashed line) and stressed (continuous line) rainbow trout. Each value is the mean ± SEM (*n* = 8/group). ^∗^*P* < 0.05 relative to Control at the same time point. Different letters indicate significant differences (*P* < 0.05) between time points within the same experimental group. Gray band indicates the dark phase of the daily cycle. The arrow indicates feeding time (ZT2).

Hepatic levels of glycogen, glucose and lactate in control and stressed trout are represented in Figure 2. Glycogen content in control fish daily oscillated, thus being higher during the day onset (ZT0) and being low during the second half of the night (ZT20). Averaged content was 156.4 ± 11.2 μmol/g tissue. Stress exposure resulted in a 20% decrease of averaged glycogen content up to 125.6 ± 7.6 μmol/g tissue, together with the loss of the daily fluctuation.

No significant daily oscillation was observed for glucose and lactate content in liver of control non-stressed trout. Averaged contents were 32.9 ± 0.7 μmol/g tissue, and 2.3 ± 0.1 μmol/g tissue, respectively. When trout were subjected to high stocking density the averaged content of each metabolite significantly decreased (glucose: 25.6 ± 0.3 μmol/g tissue; lactate: 0.8 ± 0.1 μmol/g tissue) relative to Control.

### Carbohydrate Metabolism-Related Parameters

**FIGURE 3:**
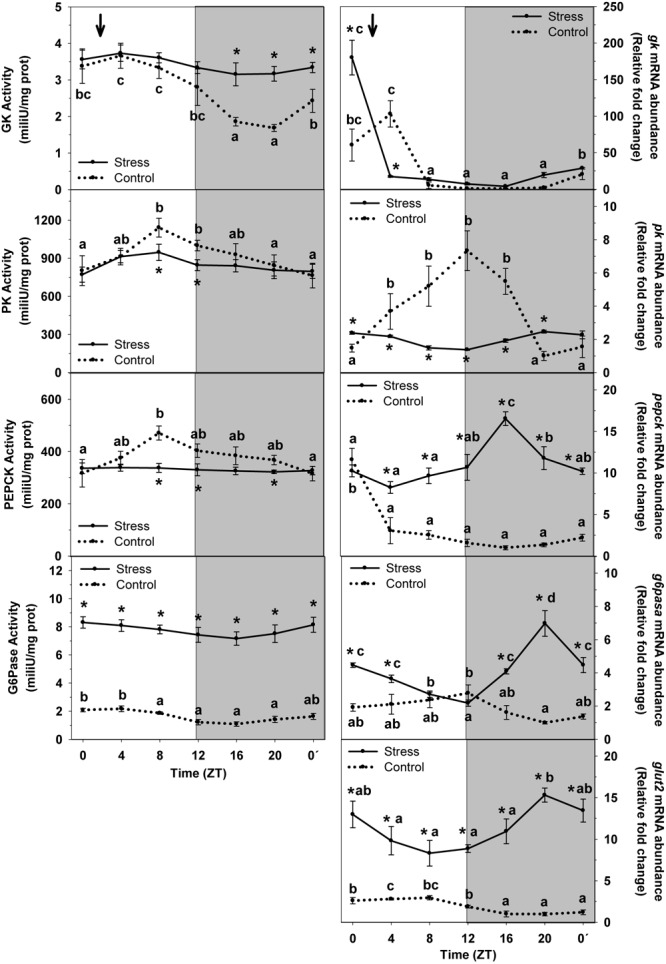
Daily profile of enzyme activity and gene expression of different parameters of hepatic carbohydrate metabolism in fish subjected at control (dashed line) and stress (continuous line) conditions. Each value is the mean ± SEM (*n* = 8/group on enzyme activity and *n* = 4/group on gene expression). ^∗^*P* < 0.05 relative to Control at the same time point. Different letters indicate significant differences (*P* < 0.05) between time points within the same experimental group. Gray band indicates the dark phase of the daily cycle. The arrow indicates feeding time (ZT2).

The daily profile of enzyme activity and mRNA abundance of carbohydrate metabolism enzymes, and glucose transporter expression (GLUT2) in trout liver of Control and Stress groups is shown in Figure 3. The *cosinor* analysis corroborated the presence of significant rhythms for most of them. Accordingly, GK activity in liver of control fish displayed a daily variation with significantly higher values during the day (ZT4) and basal levels at night (ZT20). Averaged activity along the 24-h cycle was of 2.71 mU/mg prot. Stress caused a drastic increase of averaged activity, up to 25% (3.41 mU/mg prot) relative to that of Control. Such increase was mainly due to the increase of the enzyme activity observed in stressed trout at night, which also made the daily fluctuation not to reach significance level. In addition, a significant rhythm was observed for *gk* mRNA abundance in control fish, with peaking levels during the first half of the day (ZT4). Stress caused a 4-h shift of peaking levels, which advanced to the light onset (ZT0), but also a nearly 45% increase of averaged expression (38.6 ± 11.6 relative fold change units) compared to control group (26.8 ± 8.2 relative units).

A significant daily rhythm of PK activity was noted in non-stressed fish, with the highest values being observed during the second half of the day (ZT8), thus progressively decreasing to reach basal levels at the night–day transition (ZT0′). The magnitude of the oscillation was attenuated in stressed trout, which resulted in a slight decrease of mean activity levels (845.6 ± 24.1 mU/mg prot) relative to that of Control (913.1 ± 48.6 mU/mg prot). In the same way than the activity, *pk* rhythmically expressed in Control, with peaking values at the early night (ZT12) and basal levels during the end of the night. Stress by high stocking density affected such rhythm in such a way that it was almost blunted and averaged expression was inhibited (2.1 ± 0.1 relative fold change units) to almost half of that of Control (3.7 ± 0.5 relative units).

PEPCK enzyme activity rhythmic in control group, with peaking levels occurring at ZT8, and basal levels being observed around the day onset (ZT0). Averaged activity levels were 376.2 ± 20.1 mU/mg prot. This rhythm disappeared in trout subjected to high stocking density, and a decrease of averaged enzyme activity was also noted (331.1 ± 2.4 mU/mg prot), relative to that of non-stressed fish. On the other hand, *pepck* displayed rhythmic expression in liver of control group, with peaking values during the day onset (ZT0) and basal levels at the first half of the night (ZT16). Stress exposure resulted in a dramatic alteration of this profile in such a way that averaged expression abruptly increased (11.1 ± 0.6 relative fold change units) relative to that of Control (2.5 ± 0.4 relative units), but also peaking values shifted to ZT16, thus with an 8-h advance.

G6Pase enzyme activity showed a slight daily variation in liver of control fish, with higher levels during the first half of the day (ZT4). Averaged levels were 1.7 ± 0.1 mU/mg protein. Stress exposure enhanced the enzyme activity thus making averaged levels to be higher (7.8 ± 0.2 mU/mg protein) than those of control group. However, the profile of the rhythm was not affected in stressed fish, in which peaking levels of G6Pase activity occurred during the day onset. The mRNA expression did also display a rhythmic profile in control fish, with peaking values during the early night period (ZT12) and basal levels at the late night (ZT20). Averaged expression levels were 1.8 ± 0.1 relative fold change units. Stress exposure enhanced *g6pase* expression in such a way that averaged levels raised to 4.1 ± 0.3 relative units, but also the phase of the rhythm was affected (ZT20) thus displaying an 8-h delay relative to that of Control.

Expression of *glut2* in non-stressed fish was rhythmic and peaking levels were found at day-time (ZT8). Averaged levels of mRNA abundance in this group were 2.1 ± 0.2 relative fold change units. Stress altered such profile by enhancing mRNA mean expression up to 11.4 ± 0.6 relative units, but also by phase-shifting the rhythm in such a way that it was in antiphase (peaking levels at ZT20) compared to control group.

### Lipid Metabolism-Related Enzymes

**FIGURE 4:**
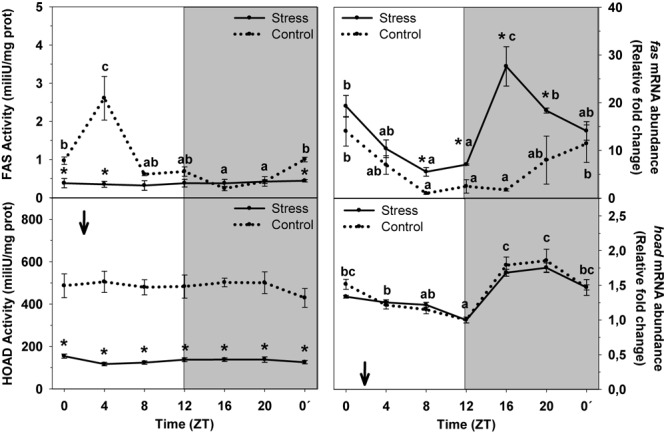
Daily profile of enzyme activity and gene expression of different parameters of hepatic lipids metabolism in fish subjected at control (dashed line) and stress (continuous line) conditions. Each value is the mean ± SEM (*n* = 8/group on enzyme activity and *n* = 4/group on gene expression). ^∗^*P* < 0.05 relative to Control at the same time point. Different letters indicate significant differences (*P* < 0.05) between time points within the same experimental group. Gray band indicates the dark phase of the daily cycle. The arrow indicates feeding time (ZT2).

Figure 4 shows daily variations of FAS and HOAD enzyme activities and mRNA abundance in liver of animals subjected or not to stress by high stocking density. FAS activity displayed a significant increase during the first half of the day (ZT4) and basal levels at night (ZT16), but the daily variation did not reach significance level. Averaged activity levels were 1.04 ± 0.13 mU/mg protein. Stress exposure blunted the daily variation, which resulted in a decrease of averaged enzyme activity to 0.37 ± 0.03 mU/mg prot, relative to control group. Control fish showed a significant rhythm of *fas* mRNA abundance. Peaking levels were noted in samples collected at the day onset (ZT0) and a minimum during the day–night transition. Averaged expression in this group was 6.5 ± 1.3 relative fold change units. Stress exposure enhanced *fas* expression, thus with averaged levels (14.6 ± 1.5 relative units) duplicating those of Control. The profile of the rhythm was also affected in such a way that two increases of *fas* abundance were observed, at ZT0, and during the night (ZT16), when basal were expected, as in Control.

HOAD enzyme activity did not show any daily variation at any experimental condition. However, stress inhibited HOAD activity, thus with averaged levels (136.7 ± 3.1 mU/mg prot) being lower than those of control group (482.7 ± 16.1 mU/mg prot). The daily rhythm of *hoad* mRNA abundance reached significance level in both experimental groups, in which peaking levels occurred during the second half of the dark period (ZT20) and basal levels at the night onset (ZT12). No differences were found between groups.

### Clock Genes Expression

**FIGURE 5:**
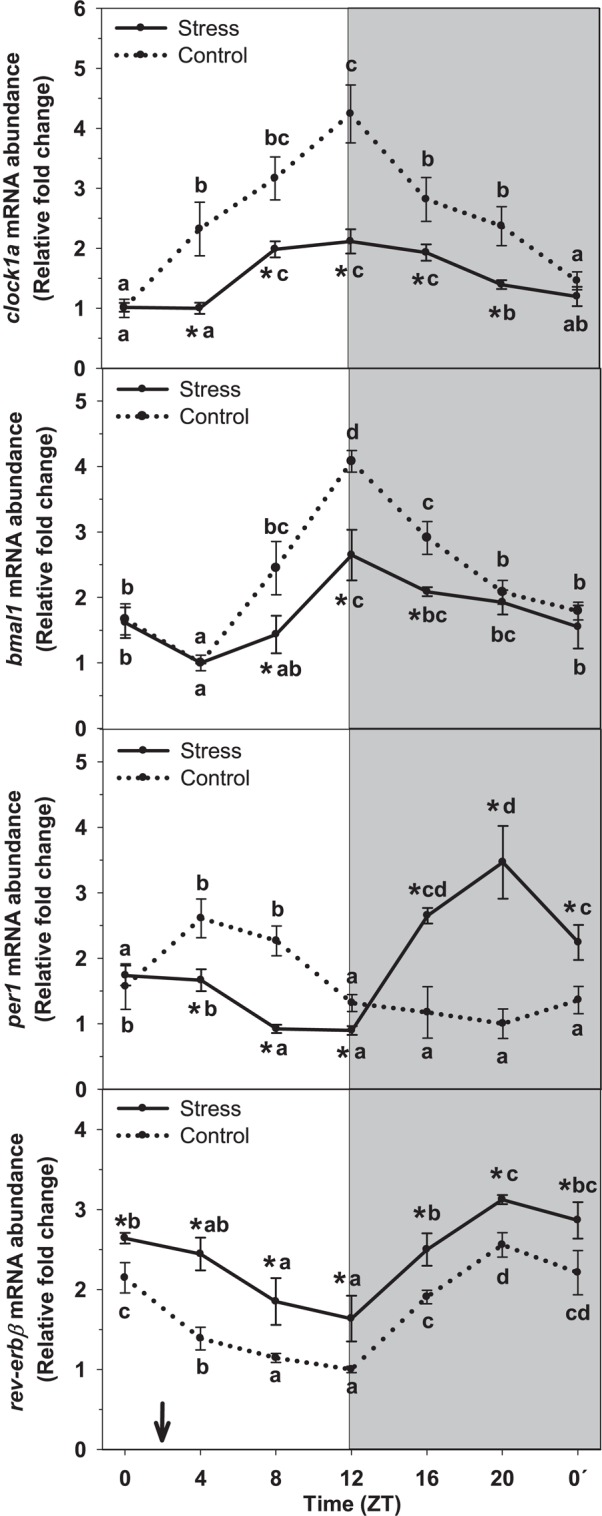
Daily profile of hepatic clock gene expression (*clock1a, bmal1, per1* and *rev-erbβ*) in fish subjected at control (dashed line) and stress (continuous line) conditions. Each value is the mean ± SEM (*n* = 4). ^∗^*P* < 0.05 relative to Control at the same time point. Different letters indicate significant differences (*P* < 0.05) between time points within the same experimental group. Gray band indicates the dark phase of the daily cycle. The arrow indicates feeding time (ZT2).

The rhythm of clock genes mRNA abundance in liver of rainbow trout and the effect of stress on such rhythms is represented on Figure 5. In control fish, the rhythm of *clock1a* was significant, and peaking values were observed during the day–night transition (ZT12), whereas basal values occurred at the day onset (ZT0). Averaged mRNA levels were 2.5 ± 0.2 relative fold change units. Stress inhibited gene expression but did not affect the profile of the rhythm, thus displaying peaking values (ZT12) in phase with that of Control. However, averaged expression decreased in stressed fish (1.5 ± 0.1 relative units) reaching a 60% of that of control group.

Similarly to that of *clock1a*, assessment of *bmal1* mRNA abundance revealed the existence of a significant rhythm with peaking values occurring at ZT12, and basal levels being observed at day-time (ZT4). Averaged expression in this group was 2.3 ± 0.2 relative fold change units. This profile was not affected when trout were subjected to stress. Then, peaking values were observed in samples collected at ZT12, as in control group. However, the inhibitory effect of stress in *bmal1* expression was noted and averaged mRNA levels decreased (1.7 ± 0.1 relative units) respect that of Control.

The daily profile of *per1* mRNA abundance in liver of control trout reached significance level, with peaking values occurring at day-time (ZT4) and basal levels during the end of the night (ZT20). Averaged mRNA levels were 1.6 ± 0.1 relative fold change units. Stress exposure affected such profile in a way that a shift of peaking levels was noted. Then, the time of the peak was observed during the second half of the night (ZT20), i.e., with an 8-h advance relative to that of Control. Averaged mRNA levels slightly increased in stressed fish, up to 1.9 ± 0.2 relative units.

By other hand, assessment of *rev-erbβ* mRNA abundance revealed the existence of a significant daily rhythm in liver of control fish. Peaking levels were found at the end of the night (ZT20), and basal levels at the end of the day (ZT12). Averaged mRNA levels were 1.8 ± 0.1 relative fold change units in this group. Stress enhanced *rev-erbβ* expression in such a way that averaged mRNA levels in stressed trout were near 33% higher (2.4 ± 0.1 relative units) than those of Control. No variation was observed for the profile of *rev-erbβ* expression in stressed fish, thus with peaking mRNA levels occurring at ZT20, and basal levels at ZT12, as in control group.

### Expression of sirt1, GR1, and GR2

**FIGURE 6:**
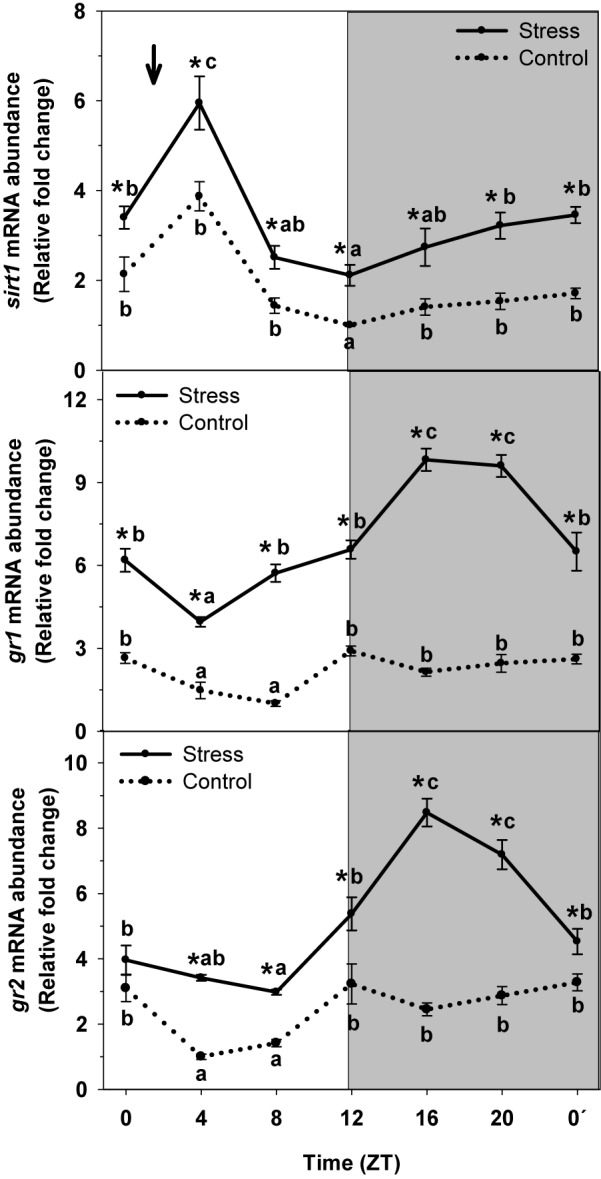
Daily expression profile of *sirt* and glucocorticoids receptors (*GR1* and *GR2*) in the liver of rainbow trout subjected at control (dashed line) and stress (continuous line) conditions. Each value is the mean ± SEM (*n* = 4). ^∗^*P* < 0.05 relative to Control at the same time point. Different letters indicate significant differences (*P* < 0.05) between time points within the same experimental group. Gray band indicates the dark phase of the daily cycle. The arrow indicates feeding time (ZT2).

To identify the role played by sirtuin1 as possible mediator of the effect of stress on liver rhythmic physiology and its interaction with cortisol as a well-known mediator of such effect, the daily profile of mRNA abundance of *sirt1* and glucocorticoid receptors (*gr1* and *gr2*) was also evaluated in trout subjected or not to stress (Figure 6). Control group exhibited a significant rhythm of *sirt1* in liver, with the peak being observed at ZT4 and a minimum during the night onset (ZT12). The Averaged mRNA abundance was 1.9 ± 0.3 relative fold change units. This profile was not affected by stress exposure, but enhances expression was noted, thus averaged levels raised to 3.3 ± 0.2 relative units in stressed trout.

Both GRs (GR1 and GR2) did exhibit a significant rhythm of mRNA abundance with high nocturnal levels and basal levels occurring at day-time independently of the experimental condition. Averaged mRNA levels for control group were 2.2 ± 0.3 relative fold change units for *gr1*, and 2.5 ± 0.3 relative units for *gr2*. Stress exposure resulted in the increase gene expression for both *gr1* and *gr2*. Then, averaged levels of mRNA abundance were 6.9 ± 0.4 relative units for *gr1*, and 5.1 ± 0.4 relative units for *gr2*, thus triplicating (*gr1*) and duplicating (*gr2*) that of Control.

## Discussion

Stress triggers in the body a series of responses in order to obtain the energy needed to deal with an adverse situation. Liver is an important source of that energy. When exposed to a stressful situation, a cascade of hormonal signaling occurs in which cortisol participates in the maintenance of the physiological response.

Our results show daily rhythm of cortisol in non-stressed fish, with peaking levels being observed at the same temporal window than feeding time. Exposure to high stocking density results in enhanced cortisol averaged levels and a significant disturbance of its daily rhythm, with peaking levels occurring at night (ZT16). These results agree with that previously reported for the same species (Gesto et al., 2013), in which cortisol levels increased shortly after animals were subjected to stress and remained elevated for several days. The daily rhythms of plasma cortisol levels have been also described in humans and rodents, and display a robust oscillation with the peak occurring shortly before the active phase initiates, morning in humans and early evening in nocturnal rodents (see rev. Koch et al., 2017). In addition, disruption of the circadian clock machinery (responsible of the daily rhythm of the hormone) associates with altered glucocorticoid concentration and daily profile, but also metabolic impairments and depression (Turek et al., 2005; Albrecht, 2010; Mukherjee et al., 2010; Barclay et al., 2012; Leliavski et al., 2014).

Complementarily, plasma glucose and lactate levels displayed a daily oscillation in control fish. Stress exposure resulted in enhanced glucose and lactate levels and altered oscillation. Such results might be expected, since stress response results in the activation of those mechanisms involved in providing nutrients all over the time a stressor is present. On the contrary, a daily oscillation was also found for glycogen, glucose, and lactate content in liver of non-stressed fish. Stress exposure resulted in a decrease of them all, leading for the oscillation to even disappear (lactate). Such results are indicative of hepatic metabolism to change in order to mobilize resources in order for the animal to cope with, and overcome the adverse situation.

### Changes in Parameters Related to Carbohydrate and Lipid Metabolism

Daily rhythms of enzyme activity and mRNA abundance are observed in most of the carbohydrate metabolism-related parameters within the liver of non-stressed trout. These rhythms are reported to be under circadian control in trout liver (Hernández-Pérez et al., 2015, 2017). Stress affects the daily oscillation of enzyme activities in a way that the amplitude of most of them decreases (GK, PK) and even disappears (PEPCK). In addition, the daily rhythm of mRNA abundance for carbohydrate metabolism-related parameters is also affected, thus increasing averaged levels (*gk, pepck, g6pase*, and *glut2*), but also displaying a phase-shifting. All these changes are consistent with increased glycogenolytic capacity (enhanced G6Pase enzyme activity and mRNA abundance) and enabled glucose transport (increased *GLUT2* abundance) that occur during stress exposure, as previously reported for by us trout subjected to acute stress (López-Patiño et al., 2014b). These results are in contrast to those reported for gilthead seabream subjected to changes in water salinity, in which a decrease in glycogenolytic capacity during chronic stress (Laiz-Carrión et al., 2003). Such discrepancy may be indicative of a species- and stress nature-specific dependence of liver physiological response to stress in fish.

Stress can induce the increased cortisol-mediated glyconeogenic capacity (Mommsen et al., 1999; Gilmour et al., 2012). In support of that, our results reveal a significant increase of mRNA abundance for the key enzyme in glucose synthesis, *pepck*, but not in the enzyme activity. However, the daily profile of both, enzyme activity and mRNA abundance, is altered in liver of stressed trout relative to that observed in control fish. Then, during the response to stress, regulatory mechanisms may exist at both transcriptional and post-transcriptional levels during stress exposure. Similar results are described for cultured trout hepatocytes (Sathiyaa and Vijayan, 2003), but also for animals subjected to different stressors (Aluru and Vijayan, 2009).

On the other hand, increased hepatic glucose production under stress conditions makes this carbohydrate available for use in glycolytic processes. Then, during stress exposure, one might expect a decrease of liver glycolytic capacity, as herein reported with decreased PK activity and mRNA abundance, together with altered daily oscillation of both. Such results agree with that previously reported for trout subjected to 60-min manipulation (Wiseman et al., 2007), or gilthead sea bream exposed to a drop of temperature (Kyprianou et al., 2010), and brook trout (*Salvelinus fontinalis*) stressed by density (Vijayan and Leatherland, 1990). All these changes occur in order to ensure glucose supply to other tissues during prolonged stress. Then, liver is important during the response to stress, since this organ mobilizes and releases glucose reserves into the bloodstream according to that needed.

With respect to lipids metabolism within the liver of rainbow trout, daily variations of mRNA abundance are observed for *fas* and *hoad*, but the respective enzyme activities do not display such oscillation. This is in agreement with our previous data describing daily rhythms of lipid metabolism-related parameter in liver of the same species (Hernández-Pérez et al., 2015, 2017), thus with the control exerted by a circadian oscillator. Stress exposure results in altered enzyme activities, but also in a change of the rhythm of *fas* abundance. Studies carried out in other teleost species describe that acute stress leads to increased plasma fatty acids levels in Atlantic salmon (Waring et al., 1992) and carp (Ruane et al., 2001). Such results together with that herein reported point to a decrease of liver lipogenic activity during stress exposure. In addition, decreased HOAD activity is indicative of increased fatty acid release from the liver into the blood. Then, when trout is subjected to stress, the liver displays changes in enzyme activities and mRNA abundance that even affect to their daily oscillation. These changes occur in order for this organ to provide energy resources that allow the whole organism to cope with the adverse situation.

### Hepatic Circadian System

Being able to anticipate daily changes in the environment is an evolutionary advantage for most species on earth. Then, organisms from plants to mammals have developed endogenous circadian oscillators that make possible for them to estimate the time of day. The alteration of the activity of such endogenous clocks results in an adverse situation for the animal, since rhythmic behavioral and physiological functions may decouple. Stress exposure is reported to negatively affect the activity of the circadian system (Takahashi et al., 2013). Glucocorticoids, as stress response mediators, play a main role in such effect in mammals, at least in peripheral locations hosting circadian oscillators such as heart, kidney or liver (Balsalobre et al., 2000). Our results in fish not only point to cortisol as mediator of the effect of stress on the circadian system at peripheral locations (herein reported), but also at the hypothalamus, where the central circadian oscillator is located (Naderi et al., 2018). In the same way, glucocorticoids administration in goldfish results in altered rhythmic functions (López-Patiño et al., 2014a; Sánchez-Bretaño et al., 2015).

With respect to our herein reported results, daily rhythms of clock genes exist in liver of rainbow trout. Those rhythms perfectly fit with those previously reported by us in the same tissue, for which the presence of a circadian oscillator was evidenced (Hernández-Pérez et al., 2017). Stressing trout by high stocking density results in decreased amplitude of *clock1a* and *bmal1* rhythms and averaged mRNA levels. However the phase of the rhythm remained similar to that of non-stressed animals. On the contrary, *per1* expression displayed both a phase-shift and a slight increase of averaged mRNA levels in stressed trout. These results agree with that previously reported for liver of mammals subjected to moderate stress, where *bmal* and *per* expression is altered (Takahashi et al., 2013). These coincidences are indicative of the circadian system to be influenced by stress, with the underlying mechanisms being apparently well conserved in vertebrates.

By other hand, *rev-erbβ* displays significant rhythms of mRNA abundance in liver of rainbow trout, which is indicative of a regulatory role played by this nuclear receptor in fish liver circadian physiology. In this way, studies carried out in mammals indicate the modulatory action of *rev-erbα* in modulating different aspects of liver metabolism (Duez and Staels, 2009), but also the inhibitory effect exerted by the nuclear receptor on *bmal1* expression (Guillaumond et al., 2005). Thus, when *rev-erb* expression increases, one may expect decreased *bmal1* mRNA abundance. This is exactly what we found in liver of trout stressed by high stocking density, where enhanced *rev-erbβ* expression was noted all over the day, thus resulting in a significant increase of averaged mRNA levels, relative to non-stressed animals. However, stress did not affect the phase of such rhythm. Then, in a stressful situation, altered hepatic energy status leads for *rev-erbβ* abundance to increase, thus inhibiting *bmal1* expression with the subsequent desynchronization of the hepatic oscillator. Such results confirm the role played by this nuclear receptor during the response of liver circadian system to stress in fish.

Cortisol appears to also mediate effects of stress on the circadian system, since the glucocorticoid enhances *per1a* and *per1b*, and inhibits *clock* and *bmal1* in liver of goldfish (Sánchez-Bretaño et al., 2016). Then, we did evaluate the rhythmic expression profile of the general glucocorticoid receptors (GR1 and GR2) and how can stress affect such profile, as long as the activation of these receptors results in altered profile of clock genes expression in mammals (see rev. Koch et al., 2017). Our results reveal significant rhythms of *gr1* and *gr2* abundance, which is indicative of the circadian system to control them in fish, as reported for mammals, in which the sensitivity of GRs is also under circadian regulation (Lamia et al., 2011). On the other side, the activation of GRs modulates the transcription of certain genes (Pratt, 1993; Jewell et al., 1995), with clock genes among them. Then, the inter-relationship between circadian system and glucocorticoids is plausible in fish. Stress by high stocking density not only enhances plasma cortisol levels (peaking at night), but also results in increased mRNA levels of *gr1* and *gr2*, with the peaks occurring at the same time (ZT16) than that of the glucocorticoid. Such enhanced expression may be responsible of the decrease of averaged *clock1a* and *bmal1* expression herein reported. At the same time, *per1* expression increases in stressed trout, and a phase-shift is also observed. Overall, a desynchronization of liver circadian physiology occur in stressed trout.

By other hand, our previous results reveal that cortisol may not be the only mediator of the effect of stress on trout circadian physiology, since treatments with the general GRs antagonist does not totally prevent such effect of stress on hypothalamic clock genes (Naderi et al., 2018). Accordingly, we evaluated the expression of *sirtuin1*, which may be considered as a link between circadian clocks and sensing of cell energy status (see rev. Delgado et al., 2017). Sirtuin1 relates to the circadian system through two main mechanisms, the first one consists on the dependence on the NAD^+^ as cofactor, which has a rhythmic biosynthesis, thus driving the rhythm of sirtuin1 activity (Nakahata et al., 2009; Ramsey et al., 2009). In addition, SIRT1 modulates the cell circadian system at central level, thus influencing the daily rhythms of *bmal1, per2* and *cry1* (Asher et al., 2010), and in peripheral tissues, such as liver (Nogueiras et al., 2012). Our results in rainbow trout agree with that above mentioned, since a daily rhythm of *sirt1* mRNA abundance is observed in trout liver of non-stressed trout, thus pointing at the interaction between sirtuin1 and circadian system to also exist in fish. By other hand, no change of the daily profile but an increase of averaged *sirt1* mRNA levels was observed in liver of stressed trout. We previously reported similar results for hypothalamus of trout exposed to identical stressing conditions (Naderi et al., 2018). In addition, our preliminary results also confirm the key role of *sirt1* during the response to stress of the circadian system, since the sirt1 inhibitor (EX527) prevents the expected variation of *clock genes* and *sirt1* expression when trout are subjected to identical stressing conditions (unpublished). However, we cannot discard the interaction between sirt1 and other mediators such as cortisol during the response to stress in rainbow trout. Further research is needed to evaluate these interactions.

In summary, our data confirm in fish that stress triggers a series of responses in the body in order to obtain energy that bring the animal the chance to deal with such adverse situation. Since liver plays an important role as source of that energy, stress also influence the physiology of this organ. Different mediators are involved in the response of liver circadian physiology to stress specially that of carbohydrate and lipid metabolism-related parameters and the circadian oscillator that locates in this organ. Among them, we confirm in fish a role for nuclear receptors (*rev-erbβ*), cortisol, and sirt1 as mediators. However, to evaluate the independent effect of each mediator or the existence and nature of any interaction among them deserve further research.

## Data Availability

The datasets generated for this study can be found in GenBank, AF053331, AF246146, AF246149, AF321816, NM_001124730.1, AY495372.1, AF266745, GQ489026.1, AF228695, AF342943.1, EZ774344.1, and NM_001124235.1.

## Ethics Statement

This study was carried out in accordance with the recommendations of Guidelines of he European Union Council (2010/63/UE) and of the Spanish Government (RD 55/2013) for the use of animals in research. Protocols were approved by the Ethics Committee at the University of Vigo.

## Author Contributions

JH-P and FN conducted the main experimental work and performed all the samples analysis. JH-P wrote the manuscript under the supervision of JM and ML-P. JS, JM, and ML-P conceived the experiments. JS and JM contributed with both reagents and goods. All the authors contributed to, and approved the manuscript.

## Conflict of Interest Statement

The authors declare that the research was conducted in the absence of any commercial or financial relationships that could be construed as a potential conflict of interest.

## References

[B1] Akiyama M. Minami Y. Kuriyama K. Shibata S. (2003). MAP kinase-dependent induction of clock gene expression by alpha 1-adrenergic receptor activation. *FEBS Lett.* 542 109–114. 12729908 10.1016/s0014-5793(03)00360-0

[B2] Albrecht U. (2010). Circadian clocks in mood-related behaviors. *Ann. Med.* 42 241–251. 20350255 10.3109/07853891003677432

[B3] Aluru N. Vijayan M. M. (2009). Stress transcriptomics in fish: a role for genomic cortisol signaling. *Gen. Comp. Endocrinol.* 164 142–150. 10.1016/j.ygcen.2009.03.020 19341738

[B4] Asher G. Gatfield D. Stratmann M. Reinke H. Dibner C. Kreppel F. (2010). SIRT1 regulates circadian clock gene expression through PER2 deacetylation. *Cell* 134 317–328. 10.1016/j.cell.2008.06.050 18662546

[B5] Aslani S. Harb M. R. Costa P. S. Almeida O. F. Sousa N. Palha J. A. (2014). Day and night: diurnal phase influences the response to chronic mild stress. *Front. Behav. Neurosci.* 8:82. 10.3389/fnbeh.2014.00082 24672446 PMC3954061

[B6] Balment R. J. Lu W. Weybourne E. Warne J. M. (2006). Arginine vasotocin a key hormone in fish physiology and behaviour: a review with insights from mammalian models. *Gen. Comp. Endocrinol.* 147 9–16. 16480986 10.1016/j.ygcen.2005.12.022

[B7] Balsalobre A. (2002). Clock genes in mammalian peripheral tissues. *Cell Tissue Res.* 309 193–199. 12111549 10.1007/s00441-002-0585-0

[B8] Balsalobre A. Brown S. A. Marcacci L. Tronche F. Kellendonk C. Reichardt H. M. (2000). Resetting of circadian time in peripheral tissues by glucocorticoid signaling. *Science* 289 2344–2347. 11009419 10.1126/science.289.5488.2344

[B9] Barclay J. L. Husse J. Bode B. Naujokat N. Meyer-Kovac J. Schmid S. M. (2012). Circadian desynchrony promotes metabolic disruption in a mouse model of shiftwork. *PLoS One* 7:e37150. 10.1371/journal.pone.0037150 22629359 PMC3357388

[B10] Bartlang M. S. Savelyev S. A. Johansson A.-S. Reber S. O. Helfrich-Förster C. Lundkvist G. B. S. (2014). Repeated psychosocial stress at night, but not day, affects the central molecular clock. *Chronobiol. Int.* 31 996–1007. 25051430 10.3109/07420528.2014.940085

[B11] Barton B. A. (2002). Stress in fishes: a diversity of responses with particular reference to changes in circulating corticosteroids. *Integr. Comp. Biol.* 42 517–525. 10.1093/icb/42.3.517 21708747

[B12] Barton B. A Iwama G. K. (1991). Physiological changes in fish from stress in aquaculture with emphasis on the response and effects of corticosteroids. *Annu. Rev. Fish Dis.* 1 3–26. 10.1016/0959-8030(91)90019-G

[B13] Barton B. A. Schreck C. B. Barton L. D. (1987). Effects of chronic cortisol administration and daily acute stress on growth, physiological conditions, and stress responses in juvenile rainbow trout. *Dis. Aquat. Organ.* 2 173–185.

[B14] Beato M. Arnemann J. Chalepakis G. Slater E. Willmann T. (1987). Gene regulation by steroid hormones. *J. Steroid Biochem.* 27 9–14. 2826895 10.1016/0022-4731(87)90288-3

[B15] Betancor M. B. McStay E. Minghetti M. Migaud H. Tocher D. R. Davie A. (2014). Daily rhythms in expression of genes of hepatic lipid metabolism in Atlantic salmon (*Salmo salar* L.). *PLoS One* 9:e106739. 10.1371/journal.pone.0106739 25184355 PMC4153669

[B16] Cerdá-Reverter J. M. Zanuy S. Carrillo M. Madrid J. A. (1998). Time-course studies on plasma glucose, insulin, and cortisol in sea bass (*Dicentrarchus labrax*) held under different photoperiodic regimes. *Physiol. Behav.* 64 245–250. 9748089 10.1016/s0031-9384(98)00048-1

[B17] Challet E. (2015). Keeping circadian time with hormones. *Diabetes Obes. Metab.* 17 76–83. 10.1111/dom.12516 26332971

[B18] Chong N. W. Chaurasia S. S. Haque R. Klein D. C. Iuvone P. M. (2003). Temporal-spatial characterization of chicken clock genes: circadian expression in retina, pineal gland, and peripheral tissues. *J. Neurochem.* 85 851–860. 12716417 10.1046/j.1471-4159.2003.01723.x

[B19] Chrousos G. P. Kino T. (2005). Intracellular glucocorticoid signaling: a formerly simple system turns stochastic. *Sci. Stke* 2005:e48. 16204701 10.1126/stke.3042005pe48

[B20] Cohen S. Vainer E. Matar M. A. Kozlovsky N. Kaplan Z. Zohar J. (2015). Diurnal fluctuations in HPA and neuropeptide Y-ergic systems underlie differences in vulnerability to traumatic stress responses at different zeitgeber times. *Neuropsychopharmacology* 40 774–790. 10.1038/npp.2014.257 25241802 PMC4289967

[B21] Conde-Sieira M. Muñoz J. L. López-Patiño M. A. Gesto M. Soengas J. L. Míguez J. M. (2014). Oral administration of melatonin counteracts several of the effects of chronic stress in rainbow trout. *Domest. Anim. Endocrinol.* 46 26–36. 10.1016/j.domaniend.2013.10.001 24411181

[B22] Coomans C. E. Lucassen E. A. Kooijman S. Fifel K. Deboer D. Rensen P. C. N. (2015). Plasticity of circadian clocks and consequences for metabolism. *Diabetes Obes. Metab.* 17 65–75. 10.1111/dom.12513 26332970

[B23] De Bosscher K. Haegeman G. (2009). Minireview: latest perspectives on anti-inflammatory actions of glucocorticoids. *Mol. Endocrinol.* 23 281–291. 10.1210/me.2008-0283 19095768 PMC5428155

[B24] Delgado M. J. Alonso-Gómez A. L. Gancedo B. De Pedro N. Valenciano A. I. Alonso Bedate M. (1993). Serotonin N-acetyltransferase (NAT) activity and melatonin levels in the frog retina are not correlated during the seasonal cycle. *Gen. Comp. Endocrinol.* 92 143–150. 8282167 10.1006/gcen.1993.1151

[B25] Delgado M. J. Cerdá-Reverter J. M. Soengas J. L. (2017). Hypothalamic integration of metabolic, endocrine, and circadian signals in fish: involvement in the control of food intake. *Front. Neurosci.* 11:354. 10.3389/fnins.2017.00354 28694769 PMC5483453

[B26] Della Ragione F. Comitato R. Angelini F. D’Esposito M. Cardone A. (2005). Molecular cloning and characterization of the clock gene period2 in the testis of lizard *Podarcis sicula* and its expression during seasonal reproductive cycle. *Gene* 19 105–112. 16289937 10.1016/j.gene.2005.08.018

[B27] Ducouret B. Tujague M. Ashraf J. Mouchel N. Servel N. Valotaire Y. (1995). Cloning of a teleost fish glucocorticoid receptor shows that it contains a deoxyribonucleic acid-binding domain different from that of mammals. *Endocrinology* 136 3774–3783. 7649084 10.1210/endo.136.9.7649084

[B28] Duez H. Staels B. (2009). Rev-erb-alpha: an integrator of circadian rhythms and metabolism. *J. Appl. Physiol.* 107 1972–1980. 10.1152/japplphysiol.00570.2009 19696364 PMC2966474

[B29] Ebbesson L. O. Björnsson B. T. Ekström P. Stefansson S. O. (2008). Daily endocrine profiles in parr and smolt Atlantic salmon. *Comp. Biochem. Physiol. A Mol. Integr. Physiol.* 151 698–704. 10.1016/j.cbpa.2008.08.017 18790069

[B30] Falcón J. (1999). Cellular circadian clocks in the pineal. *Prog. Neurobiol.* 58 121–162. 10338357 10.1016/s0301-0082(98)00078-1

[B31] Falcón J. Besseau L. Sauzet S. Boeuf G. (2007). Melatonin effects on the hypothalamo-pituitary axis in fish. *Trends Endocrinol. Metab.* 18 81–88. 17267239 10.1016/j.tem.2007.01.002

[B32] Falcón J. Migaud H. Muñoz-Cueto J. A. Carrillo M. (2010). Current knowledge on the melatonin system in teleost fish. *Gen. Comp. Endocrinol.* 165 469–482. 10.1016/j.ygcen.2009.04.026 19409900

[B33] Fonken L. K. Weber M. D. Daut R. A. Kitt M. M. Frank M. G. Watkins L. R. (2016). Stress-induced neuroinflammatory priming is time of day dependent. *Psychoneuroendocrinology* 66 82–90. 10.1016/j.psyneuen.2016.01.006 26799851 PMC4788538

[B34] Freedman L. P. (1992). Anatomy of the steroid receptor zinc finger region. *Endocr. Rev.* 13 129–145. 1618160 10.1210/edrv-13-2-129

[B35] Fujiwara T. Cherrington A. D. Neal D. N. McGuinness O. P. (1996). Role of cortisol in the metabolic response to stress hormone infusion in the conscious dog. *Metabolism* 45 571–578. 8622599 10.1016/s0026-0495(96)90026-8

[B36] Garside H. Stevens A. Farrow S. Normand C. Houle B. Berry A. (2004). Glucocorticoid ligands specify different interactions with NF-kappaB by allosteric effects on the glucocorticoid receptor DNA binding domain. *J. Biol. Chem.* 279 50050–50059. 15355994 10.1074/jbc.M407309200

[B37] Gattermann R. Weinandy R. (1996). Time of day and stress response to different stressors in experimental animals. Part I: golden hamster (*Mesocricetus auratus* Waterhouse, 1839). *J. Exp. Anim. Sci.* 38 66–76. 9226964

[B38] Gesto M. López-Patiño M. A. Hernández J. Soengas J. L. Míguez J. M. (2013). The response of brain serotonergic and dopaminergic systems to an acute stressor in rainbow trout: a time course study. *J. Exp. Biol.* 216 4435–4442. 10.1242/jeb.091751 24031060

[B39] Gilmour K. M. Kirkpatrick S. Massarsky A. Pearce B. Saliba S. Stephany C. É. (2012). The influence of social status on hepatic glucose metabolism in rainbow trout *Oncorhynchus mykiss*. *Physiol. Biochem. Zool.* 85 309–320. 10.1086/666497 22705482

[B40] Goldstein R. E. Reed G. W. Wasserman D. H. Williams P. E. Lacy D. B. Buckspan R. (1992). The effects of acute elevations in plasma cortisol levels on alanine metabolism in the conscious dog. *Metabolism* 41 1295–1303. 1461135 10.1016/0026-0495(92)90099-v

[B41] Goldstein R. E. Wasserman D. H. McGuinness O. P. Lacy D. B. Cherrington A. D. Abumrad N. N. (1993). Effects of chronic elevation in plasma cortisol on hepatic carbohydrate metabolism. *Am. J. Physiol.* 264 E119–E127. 8430780 10.1152/ajpendo.1993.264.1.E119

[B42] Gooley J. J. Lu J. Chou T. C. Scammell T. E. Saper C. B. (2001). Melanopsin in cells of origin of the retinohypothalamic tract. *Nat. Neurosci.* 4:1165. 10.1038/nn768 11713469

[B43] Groeneweg F. L. Karst H. de Kloet E. R. Joëls M. (2012). Mineralocorticoid and glucocorticoid receptors at the neuronal membrane, regulators of nongenomic corticosteroid signalling. *Mol. Cell. Endocrinol.* 350 299–309. 10.1016/j.mce.2011.06.020 21736918

[B44] Guillaumond F. Dardente H. Giguère V. Cermakian N. (2005). Differential control of Bmal1 circadian transcription by REV-ERB and ROR nuclear receptors. *J. Biol. Rhythms* 20 391–403. 16267379 10.1177/0748730405277232

[B45] Halberg F. Reinberg A. (1967). Rythmes circadiens et rythmes de basse fréquence en physiologie humaine. *J. Physiol.* 59 117–200. 5006830

[B46] Hardin P. E. Panda S. (2013). Circadian timekeeping and output mechanisms in animals. *Curr. Opin. Neurobiol.* 23 724–731. 10.1016/j.conb.2013.02.018 23731779 PMC3973145

[B47] Hernández-Pérez J. Míguez J. M. Librán-Pérez M. Otero-Rodiño C. Naderi F. Soengas J. L. (2015). Daily rhythms in activity and mRNA abundance of enzymes involved in glucose and lipid metabolism in liver of rainbow trout, *Oncorhynchus mykiss*. Influence of light and food availability. *Chronobiol. Int.* 32 1391–1408. 10.3109/07420528.2015.1100633 26587750

[B48] Hernández-Pérez J. Míguez J. M. Naderi F. Soengas J. L. López-Patiño M. A. (2017). Influence of light and food on the circadian clock in liver of rainbow trout, *Oncorhynchus mykiss*. *Chronobiol. Int.* 34 1259–1272. 10.1080/07420528.2017.1361435 28933632

[B49] Isorna E. De Pedro N. Valenciano A. I. Alonso-Gómez A. L. Delgado M. J. (2017). Interplay between the endocrine and circadian systems in fishes. *J. Endocrinol.* 232 141–159. 10.1530/JOE-16-0330 27999088

[B50] Jaillon O. Aury J. M. Brunet F. Petit J. L. Stange-Thomann N. Mauceli E. (2004). Genome duplication in the teleost fish *Tetraodon nigroviridis* reveals the early vertebrate proto-karyotype. *Nature* 431 946–957. 15496914 10.1038/nature03025

[B51] Jewell C. M. Webster J. C. Burnstein K. L. Sar M. Bodwell J. E. Cidlowski J. A. (1995). Immunocytochemical analysis of hormone mediated nuclear translocation of wild type and mutant glucocorticoid receptors. *J. Steroid Biochem. Mol. Biol.* 55 135–146. 7495692 10.1016/0960-0760(95)00174-x

[B52] Kaneko M. Hernandez-Borsetti N. Cahill G. M. (2006). Diversity of zebrafish peripheral oscillators revealed by luciferase reporting. *Proc. Natl. Acad. Sci. U.S.A.* 103 14614–14619. 16973754 10.1073/pnas.0606563103 PMC1600008

[B53] Kino T. Chrousos G. P. (2004). Glucocorticoid and mineralocorticoid receptors and associated diseases. *Essays Biochem.* 40 137–155. 15242344 10.1042/bse0400137

[B54] Koch C. E. Leinweber B. Drengberg B. C. Blaum C. Oster H. (2017). Interaction between circadian rhythms and stress. *Neurobiol. Stress* 6 57–67. 28229109 10.1016/j.ynstr.2016.09.001 PMC5314421

[B55] Kondratov R. V. (2007). A role of the circadian system and circadian proteins in aging. *Ageing Res. Rev.* 6 12–27. 17369106 10.1016/j.arr.2007.02.003

[B56] Kyprianou T. D. Pörtner H. O. Anestis A. Kostoglou B. Feidantsis K. Michaelidis B. (2010). Metabolic and molecular stress responses of gilthead seam bream *Sparus aurata* during exposure to low ambient temperature: an analysis of mechanisms underlying the winter syndrome. *J. Comp. Physiol. B* 180 1005–1018. 10.1007/s00360-010-0481-y 20514487

[B57] Laiz-Carrión R. Martín Del Río M. P. Miguez J. M. Mancera J. M. Soengas J. L. (2003). Influence of cortisol on osmoregulation and energy metabolism in gilthead seabream *Sparus aurata*. *J. Exp. Zool. A Comp. Exp. Biol.* 298 105–118. 12884272 10.1002/jez.a.10256

[B58] Lamia K. A. Papp S. J. Yu R. T. Barish G. D. Uhlenhaut N. H. Jonker J. W. (2011). Cryptochromes mediate rhythmic repression of the glucocorticoid receptor. *Nature* 480 552–556. 10.1038/nature10700 22170608 PMC3245818

[B59] Leliavski A. Shostak A. Husse J. Oster H. (2014). Impaired glucocorticoid production and response to stress in Arntl-deficient male mice. *Endocrinology* 155 133–142. 10.1210/en.2013-1531 24189141

[B60] Librán-Pérez M. Polakof S. López-Patiño M. A. Míguez J. M. Soengas J. L. (2012). Evidence of a metabolic fatty acid-sensing system in the hypothalamus and Brockmann bodies of rainbow trout: implications in food intake regulation. *Am. J. Physiol. Regul. Integr. Comp. Physiol.* 302 R1340–R1350. 10.1152/ajpregu.00070.2012 22496361

[B61] López-Patiño M. A. Gesto M. Conde-Sieira M. Soengas J. L. Míguez J. M. (2014a). Stress inhibition of melatonin synthesis in the pineal organ of rainbow trout (*Oncorhynchus mykiss*) is mediated by cortisol. *J. Exp. Biol.* 217 1407–1416. 10.1242/jeb.087916 24436377

[B62] López-Patiño M. A. Hernández-Pérez J. Gesto M. Librán-Pérez M. Míguez J. M. Soengas J. L. (2014b). Short-term time course of liver metabolic response to acute handling stress in rainbow trout, *Oncorhynchus mykiss*. *Comp. Biochem. Physiol. A* 168 40–49. 10.1016/j.cbpa.2013.10.027 24239669

[B63] López-Patiño M. A. Rodríguez-Illamola A. Conde-Sieira M. Soengas J. L. Míguez J. M. (2011). Daily rhythmic expression patterns of clock1a, bmal1, and per1 genes in retina and hypothalamus of the rainbow trout, *Oncorhynchus mykiss*. *Chronobiol. Int.* 28 381–389. 10.3109/07420528.2011.566398 21721853

[B64] McEwen B. S. (2008). Central effects of stress hormones in health and disease: understanding the protective and damaging effects of stress and stress mediators. *Eur. J. Pharmacol.* 583 174–185. 10.1016/j.ejphar.2007.11.071 18282566 PMC2474765

[B65] McEwen B. S. Bowles N. P. Gray J. D. Hill M. N. Hunter R. G. Karatsoreos I. N. (2015). Mechanisms of stress in the brain. *Nat. Neurosci.* 18 1353–1363. 26404710 10.1038/nn.4086 PMC4933289

[B66] Menaker M. Moreira L. F. Tosini G. (1997). Evolution of circadian organization in vertebrates. *Braz. J. Med. Biol. Res.* 30 305–313. 9246228 10.1590/s0100-879x1997000300003

[B67] Mommsen T. P. Vijayan M. M. Moon T. W. (1999). Cortisol in teleosts: dynamics, mechanisms of action, and metabolic regulation. *Rev. Fish Biol. Fish.* 9 211–268.

[B68] Moore R. Y. Speh J. C. Patrick C. J. (1995). The retinohypothalamic tract originates from a distinct subset of retinal ganglion cells. *J. Comp. Neurol.* 352 351–366. 7706557 10.1002/cne.903520304

[B69] Mühlbauer E. Wolgast S. Finckh U. Peschke D. Peschke E. (2004). Indication of circadian oscillations in the rat pancreas. *FEBS Lett.* 564 91–96. 15094047 10.1016/S0014-5793(04)00322-9

[B70] Mukherjee S. Coque L. Cao J. L. Kumar J. Chakravarty S. Asaithamby A. (2010). Knockdown of Clock in the ventral tegmental area through RNA interference results in a mixed state of mania and depression-like behavior. *Biol. Psychiatry* 68 503–511. 10.1016/j.biopsych.2010.04.031 20591414 PMC2929276

[B71] Nader N. Chrousos G. P. Kino T. (2010). Interactions of the circadian CLOCK system and the HPA axis. *Trends Endocrinol. Metab.* 21 277–286. 10.1016/j.tem.2009.12.011 20106676 PMC2862789

[B72] Naderi F. Hernández-Pérez J. Chivite M. Soengas J. L. Míguez J. M. López-Patiño M. A. (2018). Involvement of cortisol and sirtuin1 during the response to stress of hypothalamic circadian system and food intake-related peptides in rainbow trout, *Oncorhynchus mykiss*. *Chronobiol. Int.* 35 1122–1141. 10.1080/07420528.2018.1461110 29737878

[B73] Nakahata Y. Sahar S. Astarita G. Kaluzova M. Sassone-Corsi P. (2009). Circadian control of the NAD+ salvage pathway by CLOCK-SIRT1. *Science* 324 654–657. 10.1126/science.1170803 19286518 PMC6501775

[B74] Nogueiras R. Habegger K. M. Chaudhary N. Finan B. Banks A. S. Dietrich M. O. (2012). Sirtuin 1 and sirtuin 3: physiological modulators of metabolism. *Physiol. Rev.* 92 1479–1514. 10.1152/physrev.00022.2011 22811431 PMC3746174

[B75] Panda S. Antoch M. P. Miller B. H. Su A. I. Schook A. B. Straume M. (2002). Coordinated transcription of key pathways in the mouse by the circadian clock. *Cell* 109 307–320. 12015981 10.1016/s0092-8674(02)00722-5

[B76] Paredes J. F. Vera L. M. Martinez-Lopez F. J. Navarro I. Sánchez-Vázquez F. J. (2014). Circadian rhythms of gene expression of lipid metabolism in Gilthead Sea bream liver: synchronisation to light and feeding time. *Chronobiol. Int.* 31 613–626. 10.3109/07420528.2014.881837 24517141

[B77] Pavlidis M. Greenwood L. Paalavuo M. Mölsä H. Laitinen J. T. (1999). The effect of photoperiod on diel rhythms in serum melatonin, cortisol, glucose, and electrolytes in the common dentex, *Dentex dentex*. *Gen. Comp. Endocrinol.* 113 240–250. 10082626 10.1006/gcen.1998.7190

[B78] Peirson S. N. Butler J. N. Duffield G. E. Takher S. Sharma P. Foster R. G. (2006). Comparison of clock gene expression in SCN, retina, heart, and liver of mice. *Biochem. Biophys. Res. Commun.* 351 800–807. 17092486 10.1016/j.bbrc.2006.10.118

[B79] Pfaffl M. W. (2001). A new mathematical model for relative quantification in real-time RTPCR. *Nucleic Acids Res.* 29 2002–2007. 10.1093/nar/29.9.e45 PMC55695 11328886

[B80] Polakof S. Míguez J. M. Soengas J. L. (2007). Daily changes in parameters of energy metabolism in liver, white muscle, and gills of rainbow trout: dependence on feeding. *Comp. Biochem. Physiol. A Mol. Integr. Physiol.* 147 363–374. 17317250 10.1016/j.cbpa.2007.01.009

[B81] Polakof S. Míguez J. M. Soengas J. L. (2008). Changes in food intake and glucosensing function of hypothalamus and hindbrain in rainbow trout subjected to hyperglycemic or hypoglycemic conditions. *J. Comp. Physiol. A* 194 829–839. 10.1007/s00359-008-0354-y 18663455

[B82] Portaluppi F. Smolensky M. H. Touitou Y. (2010). Ethics and methods for biological rhythm research on animals and human beings. *Chronobiol. Int.* 27 1911–1929. 10.3109/07420528.2010.516381 20969531

[B83] Pratt W. B. (1993). The role of heat shock proteins in regulating the function, folding, and trafficking of the glucocorticoid receptor. *J. Biol. Chem.* 268 21455–21458. 8407992

[B84] Ramsey K. M. Yoshino J. Brace C. S. Abrassart D. Kobayashi Y. Marcheva B. (2009). Circadian clock feedback cycle through NAMPT-mediated NAD+ biosynthesis. *Science* 324 651–654. 10.1126/science.1171641 19299583 PMC2738420

[B85] Retana-Marquez S. Bonilla-Jaime H. Vazquez-Palacios G. Dominguez-Salazar E. Martinez-Garcia R. Velazquez-Moctezuma J. (2003). Body weight gain and diurnal differences of corticosterone changes in response to acute and chronic stress in rats. *Psychoneuroendocrinology* 28 207–227. 12510013 10.1016/s0306-4530(02)00017-3

[B86] Ruane N. M. Huisman E. A. Komen J. (2001). Plasma cortisol and metabolite level profiles in two isogenic strains of common carp during confinement. *J. Fish Biol.* 59 1–12.

[B87] Samarasinghe R. A. Witchell S. F. DeFranco D. B. (2012). Cooperativity and complementarity: synergies in non-classical and classical glucocorticoid signaling. *Cell Cycle* 11 2819–2827. 10.4161/cc.21018 22801547 PMC3419059

[B88] Sánchez-Bretaño A. Alonso-Gómez A. L. Delgado M. J. Isorna E. (2016). Clock genes expression rhythms in liver of goldfish in vivo and in vitro. Possible role of glucocorticoids as a synchronizer. *J. Comp. Physiol. B* 186 73–82. 26433649 10.1007/s00360-015-0936-2

[B89] Sánchez-Bretaño A. Callejo M. Montero M. Alonso-Gómez Á. L. Delgado M. J. Isorna E. (2015). Performing a hepatic timing signal: glucocorticoids induce gper1a and gper1b expression and repress gclock1a and gbmal1a in the liver of goldfish. *J. Comp. Physiol. B* 186 73–82. 10.1007/s00360-015-0936-2 26433649

[B90] Sargin D. (2018). The role of the orexin system in stress response. *Neuropharmacology* 10.1016/j.neuropharm.2018.09.034 [Epub ahead of print]. 30266600

[B91] Sathiyaa R. Vijayan M. M. (2003). Autoregulation of glucocorticoid receptor by cortisol in rainbow trout hepatocytes. *Am. J. Physiol. Cell Physiol.* 284 C1508–C1515. 12584114 10.1152/ajpcell.00448.2002

[B92] Scheinman R. I. Gualberto A. Jewell C. M. Cidlowski J. A. Baldwin A. S. (1995). Characterization of mechanisms involved in transrepression of NF-kappa B by activated glucocorticoid receptors. *Mol. Cell. Biol.* 15 943–953. 7823959 10.1128/mcb.15.2.943 PMC231982

[B93] Shonkoff J. P. Boyce W. T. McEwen B. S. (2009). Neuroscience, molecular biology, and the childhood roots of health disparities. *JAMA* 301 2252–2259. 10.1001/jama.2009.754 19491187

[B94] Small B. C. (2005). Effect of fasting on nychthemeral concentrations of plasma growth hormone (GH), insulin-like growth factor I (IGF-I), and cortisol in channel catfish (*Ictalurus punctatus*). *Comp. Biochem. Phys. B* 142 217–223. 16126422 10.1016/j.cbpb.2005.07.008

[B95] So A. Y. L. Bernal T. U. Pillsbury M. L. Yamamoto K. R. Feldman B. J. (2009). Glucocorticoid regulation of the circadian clock modulates glucose homeostasis. *Proc. Natl. Acad. Sci. U.S.A.* 106 17582–17587. 10.1073/pnas.0909733106 19805059 PMC2757402

[B96] Spieler R. E. Noeske T. A. (1984). Effects of photoperiod and feeding schedule on diel variations of locomotor activity, cortisol, and thyroxine in goldfish. *Trans. Am. Fish. Soc.* 113 528–539.

[B97] Surjit M. Ganti K. P. Mukherji A. Ye T. Hua G. Metzger D. (2011). Widespread negative response elements mediate direct repression by agonist-liganded glucocorticoid receptor. *Cell* 145 224–241. 10.1016/j.cell.2011.03.027 21496643

[B98] Tahara Y. Shiraishi T. Kikuchi Y. Haraguchi A. Kuriki D. Sasaki H. (2015). Entrainment of the mouse circadian clock by subacute physical and psychological stress. *Sci. Rep.* 5:11417. 10.1038/srep11417 26073568 PMC4466793

[B99] Takahashi K. Yamada T. Tsukita S. Kaneko K. Shirai Y. Munakata Y. (2013). Chronic mild stress alters circadian expressions of molecular clock genes in the liver. *Am. J. Physiol. Endocrinol. Metab.* 304 E301–E309. 10.1152/ajpendo.00388.2012 23211520

[B100] Teitsma C. A. Anglade I. Toutirais G. Muñoz-Cueto J. A. Saligaut D. Ducouret B. (1998). Immunohistochemical localization of glucocorticoid receptors in the forebrain of the rainbow trout (*Oncorhynchus mykiss*). *J. Comp. Neurol.* 401 395–410. 9811116

[B101] Teitsma C. A. Bailhache T. Tujague M. Balment R. J. Ducouret B. Kah O. (1997). Distribution and expression of glucocorticoid receptor mRNA in the forebrain of the rainbow trout. *Neuroendocrinology* 66 294–304. 9349664 10.1159/000127251

[B102] Tort L. Teles M. (2011). “Endocrinology of stress. A comparative view”, in *Endocrinology* Book. 3 ed. Akin F. (Rijeka: Intech Publisher).

[B103] Turek F. W. Joshu C. Kohsaka A. Lin E. Ivanova G. McDearmon E. (2005). Obesity and metabolic syndrome in circadian Clock mutant mice. *Science* 308 1043–1045. 15845877 10.1126/science.1108750 PMC3764501

[B104] Vallone D. Frigato E. Vernesi C. Foà A. Foulkes N. S. Bertolucci C. (2007). Hypothermia modulates circadian clock gene expression in lizard peripheral tissues. *Am. J. Physiol. Regul. Integr. Comp. Physiol.* 292 160–166. 16809482 10.1152/ajpregu.00370.2006

[B105] Velarde E. Haque R. Iuvone P. M. Azpeleta C. Alonso-Gómez A. L. Delgado M. J. (2009). Circadian clock genes of goldfish, *Carassius auratus*: cDNA cloning and rhythmic expression of period and cryptochrome transcripts in retina, liver, and gut. *J. Biol. Rhythms* 24 104–113. 10.1177/0748730408329901 19346448 PMC2666933

[B106] Vijayan M. M. Ballantyne J. S. Leatherland J. F. (1991). Cortisol-induced changes in some aspects of the intermediary metabolism of *Salvelinus fontinalis*. *Gen. Comp. Endocrinol.* 82 476–486. 1652533 10.1016/0016-6480(91)90323-x

[B107] Vijayan M. M. Leatherland J. F. (1990). High stocking density affects cortisol secretion and tissue distribution in brook charr, *Salvelinus fontinalis*. *J. Endocrinol.* 124 311–318. 2155988 10.1677/joe.0.1240311

[B108] Vijayan M. M. Pereira C. Moon T. W. (1994). Hormonal stimulation of hepatocyte metabolism in rainbow trout following an acute handling stress. *Comp. Biochem. Physiol.* 108C 321–329.

[B109] Waring C. P. Stagg R. M. Poxton M. G. (1992). The effects of handling on flounder (*Platichthys flesus* L.) and Atlantic salmon (*Salmo salar* L.). *J. Fish Biol.* 41 131–144.

[B110] Weaver D. R. (1998). The suprachiasmatic nucleus: a 25-year retrospective. *J. Biol. Rhythms* 13 100–112. 9554572 10.1177/074873098128999952

[B111] Wendelaar-Bonga S. E. (1997). The stress response in fish. *Physiol. Rev.* 77 591–625. 9234959 10.1152/physrev.1997.77.3.591

[B112] Winberg S. Nilsson G. E. (1993). Roles of brain monoamine neurotransmitters in agonistic behaviour and stress reactions, with particular reference to fish. *Comp. Biochem. Physiol.* 106C 597–614.

[B113] Wiseman S. Osachoff H. Bassett E. Malhotra J. Bruno J. Vanaggelen G. (2007). Gene expression pattern in the liver during recovery from an acute stressor in rainbow trout. *Comp. Biochem. Physiol. Part D Genomics Proteomics* 2 234–244. 10.1016/j.cbd.2007.04.005 20483297

[B114] Zvonic S. Ptitsyn A. A. Conrad S. A. Scott L. K. Floyd Z. E. Kilroy G. (2006). Characterization of peripheral circadian clocks in adipose tissues. *Diabetes Metab. Res. Rev.* 55 962–970. 16567517 10.2337/diabetes.55.04.06.db05-0873

